# To a Question on the Mechanism of the Antimicrobial Action of Ortho-Benzoic Sulfimide

**DOI:** 10.3390/ph13120461

**Published:** 2020-12-13

**Authors:** Ekaterina Y. Kasap, Dmitry V. Grishin

**Affiliations:** Institute of Biomedical Chemistry (IBMC), Department of Research Laboratories, 119121 Moscow, Russia; biogene@bk.ru

**Keywords:** *o-*benzoic sulfimide, saccharin, sulfanilamide, folic acid, antimicrobial action

## Abstract

The article summarizes and compares data on the properties and biological activity of *o*-benzoic sulfimide and sulfanilamide compounds. Attention is given to the biochemical conditions under which *o-*benzoic sulfimide and sulfanilamides have similar activity groups. The results of the experimental and theoretical studies aimed at understanding the molecular organization and biological activity of folic acid and its homologous complexes are analyzed. A hypothesis about the possible mechanisms of the formation of such complexes with the participation of *o-*benzoic sulfimide is presented. The perspectives for the use of *o-*benzoic sulfimide and its homologues in biomedicine are evaluated.

## 1. Introduction

**Figure 1:**
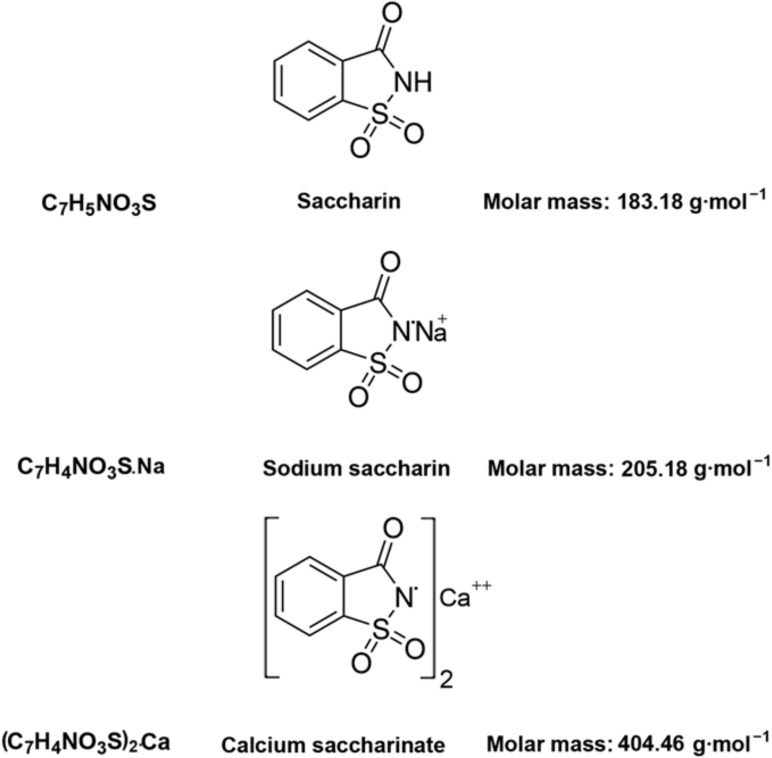
Structural and molecular formulas and relative molecular mass of saccharin and its derivatives.

**Figure 2:**
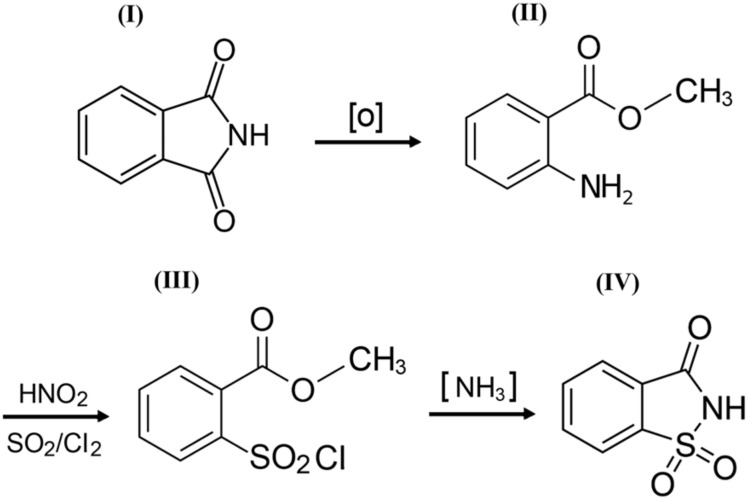
A simplified scheme of the sequence of chemical reactions in the conversion of phthalimide (**I**) to *o*-benzoic sulfimide (**IV**).

*o*-Benzoic sulfimide (1,1-dioxo-1,2-benzothiazol-3-one), better known as saccharin (Figure 1), was accidentally discovered by a chemist Constantin Fahlberg in 1879 when studying the oxidation of *o-*toluenesulfonamide for work on coal tar derivatives in the laboratory of Prof. Ira Remsen at Johns Hopkins University [1]. At this point, scientists came to understand that the crystalline hydrate of saccharin is a hundred times as sweet as sucrose (table sugar) and, for this reason, the future of this substance was associated with medicine and dietology [2,3,4,5]. A method for manufacturing saccharin was patented in 1884–1885 [6]. Initially, Fahlberg obtained saccharin from toluene by sulfation with sulfurochloridic acid. The resulting acid chloride was converted into an amide that was oxidized with potassium permanganate. Unfortunately, this method was not effective enough. In the 1950s, O. Senn and G. F. Schlaudecker developed an alternative approach that began with methyl anthranilate, which was easily procured from phthalimide or phthalic anhydride (Figure 2). Its diazotization and serial treatment with sulfur dioxide, chlorine, and finally ammonia led directly to saccharin [7].

*o*-Benzoic sulfimide gained popularity as a sugar substitute for diabetics in the 1960s when the Maumee Chemical Company of Toledo, Ohio, USA adapted Senn and Schlaudecker’s method for commercial application, allowing for the production of saccharin on an industrial scale. The major advantage of this new method was the avoidance of the costly separation of ortho- and para-isomers [7].

At the same time, the side effects of this chemical compound began to be discussed. As a result of a number of studies in the 1970s, concern arose over the carcinogenic properties of saccharin [8,9,10,11,12] such that a number of countries restricted its use in the food industry [13]. However, these assumptions were later refuted, since the incidence of bladder cancer in laboratory mice increased only if the amount of saccharin consumed significantly exceeded the acceptable dosage [14,15,16]. Similar studies found sodium saccharin did not increase the mutant frequency in the liver or bladder of the rats. These results added further support to the existence of a non-genotoxic mechanism of carcinogenic action for sodium saccharin in mice [11]. After subsequent studies failed to confirm a link between *o*-benzoic sulfimide and bladder cancer in humans, saccharin was removed from the list of potential carcinogens in the year 2000 [17]. The current United States Food and Drug Administration (FDA) confirms the acceptable daily intake (ADI) of 15 milligrams per kilogram body weight per day (mg/kg bw/d) as safe in humans [18,19,20,21,22,23,24,25,26,27,28].

During the same period, studies revealed a relationship between gut microbiome perturbed by saccharin and the expression of pro-inflammatory cytokine-inducible nitric oxide synthase (iNOS) and tumor necrosis factor (TNF-α) in the liver [19,20,21,22,23,24,25,26,27,28]. Subsequent studies have shown that *o-*benzoic sulfimide and its derivatives in concentrations from 1 to 320 mg/mL may have different inhibitory efficiencies on different bacterial species in vitro and in vivo [20,21,22,23,24,28,29]. For example, it was shown that 5 mmol of saccharin exhibits statistically significant bacteriostatic effects on *Staphylococcus aureus, Klebsiella pneumonia,* and *Pseudomonas aeruginosa*, but results in a complete abrogation of bacterial growth in *Bacillus cereus* [28].

However, despite the importance of this information, the antibacterial properties of saccharin and its homologues have not been closely studied because a mechanism for the action of these compounds on microbial cells has not yet been proposed.

Thus, this article generalizes and compares the data on the structural organization and biological activity of saccharin, searching for the most probable targets for its antimicrobial action and modeling the possible mechanism of such action at the molecular level.

## 2. *o*-Benzoic Sulfimide: Physical and Chemical Properties, Synthesis, and Structural Organization

A better understanding of the molecular mechanism underlying the possible pharmacological effect of *o*-benzoic sulfimide can be found in its physical and chemical properties. 

From a physicochemical point of view, saccharin is a colorless to white powder crystallizing in a monoclinic crystal system with a density of 0.828 g/cm^3^ [30,31,32]. The melting point is usually close to 228 °C, but when in its salt form, the melting point can exceed 300 °C [32,33]. This substance is used industrially both in the form of acid and in the form of salt. The acid form is poorly soluble in water and diethyl ether (2 g/L at 20 °C) and slightly more soluble in ethanol and acetone [31,32,33], so industries are more likely to use calcium and sodium salts, which are easily soluble in polar solvents (370 g/L and 1000 g/L at 20 °C, respectively) (Figure 1) [32]. 

From an organoleptic point of view, benzoic sulfimide solutions have a high sugar identity; saccharin, on average, is 300 times sweeter than sucrose [2]. The LD50 for this substance is 1280 mg/kg when it is administered orally to rats [34].

From a structural point of view, a molecule of saccharin is a derivative of the isoindole-1,3-dione (phthalimide (I)). For this reason, methods for the synthesis of *o*-benzoic sulfimide are based primarily on the reaction of converting phthalimide to methyl anthranilate (II), followed by its conversion to *o*-benzoic sulfimide (IV) through the formation of an intermediate called methyl 2 - (chlorosulfonyl) benzoate (III) by stepwise interaction with nitrous acid, sulfur dioxide, chlorine, and ammonia [30] (Figure 2).

## 3. Mechanism of the Biogenic Transformation of Saccharin 

In order to understand the mechanism of the antimicrobial action of saccharin, it is necessary to start with the fact that the cyclic form of *o*-benzoic sulfimide does not have active radicals that could help it quickly engage in crucial metabolic processes in pro- and eukaryotic cells. This leads to the conclusion that the cyclic form of saccharin is probably a prodrug that needs to be bioactivated. The target for such activation can be the substituted isothiazolidine ring, since it is known that substituted thiazolidines have a high hydrolysis lability in contrast to their non-substituted analogues [35,36]. The known data on the enzymatic and chemical modification of imide, lactam, and other similar cycles under extreme pH values can serve as indirect confirmation of the possibility of such a transformation [37,38,39,40,41,42]. Special attention should also be paid to reports on the use of saccharin as an alternative reagent for Gabriel synthesis (synthesis of primary amines). What is interesting is not so much the reaction itself, but its initial stages, during which, apparently, the alkaline opening of the heterocyclic compound within the structure of saccharin and its closest homologues occurs [43,44,45].

When entering the cells of pro- and eukaryotes, saccharin may be affected by high pH values and by a whole complex of enzymes, including the cytochrome P450 superfamily (CYPs; EC 1.14.14.1), which catalyzes the oxidation of the compounds with aliphatic or aromatic rings [45,46,47,48,49,50,51,52,53]. Given this, it is obvious that the isothiazolidinic ring of *o*-benzoic sulfimide can undergo chemical and/or enzymatic decyclization. The resulting radicals turn *o*-benzoic sulfimide into an activated form (XII) similar in many ways to the derivatives of *p*-amino benzenesulfonamide (sulfanilamides) and with the full range of inherent properties of these compounds. The proposed mechanism of such modification is shown in Figure 5, stage 1.

## 4. Folic Acid: A Unique Therapeutic Target

**Figure 3:**
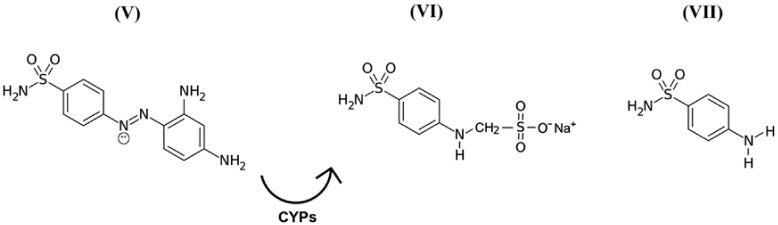
Structural and molecular formulas of historically first forms of sulfanilamide pharmaceuticals.

While discussing this topic, it is important to remember that in the 1940s, scientists discovered the precursors of a representative group of antimicrobial drugs containing *p-*aminobenzenesulfonamidic pharmacophore that could affect streptococcal bacteria [54,55], the most striking representative of which is red streptocide (Figure 3V). It was later discovered that this substance is not a drug, but rather a prodrug. The active principle was discovered to be its metabolite called sodium, (4-sulfamoylanilino) methanesulfonate (Figure 3VI). This substance is a modified water-soluble form of white streptocide (Figure 3VII), which was later obtained in its pure state [54,56].

**Figure 4:**
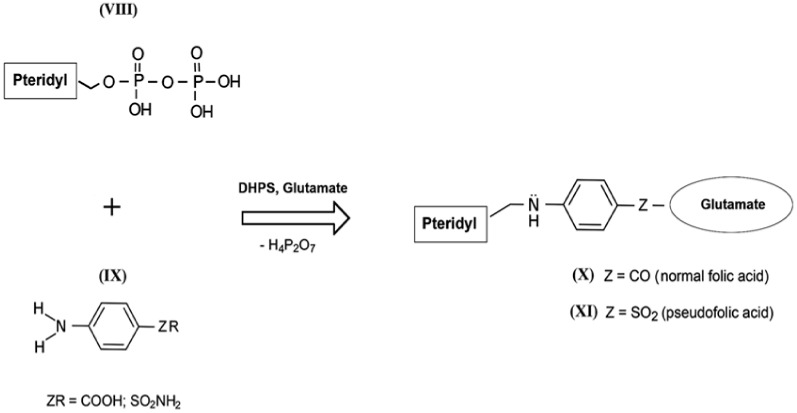
Simplified scheme of biosynthesis of normal folic acid (X) and pseudofolic acid (XI) (partly adapted from [54]).

The mechanism of the antibacterial action of sulfanilamides was studied in detail in subsequent years. In their development, prokaryotic cells, in contrast to higher eukaryotes, are capable of independent de novo synthesis of folic acid. Folic acid (folate) belongs to the Bc vitamin group that forms the structural basis of the cofactors that are mandatory participants in the transfer and transformation of single-carbon groups in the biosynthesis of nucleic acids and amino acids [57,58]. The structure of normal folate contains a residue of 2-amino-4-hydroxy-6-pyrophosphoryl-methylpteridine (Figure 4, VIII), a residue of l-glutamic acid (glutamate), and a fragment of *p*-aminobenzoic acid (Figure 4, IX) [59]. Since the enzyme that performs folic acid biosynthesis in a microbial cell (dihydropteroate synthase, DHPS) acts non-specifically during such assembly, sulfanilamides enter into intense competition with *p*-aminobenzoic acid for the active center of this enzyme [60,61,62]. As a result, a mimetic of aminobenzoic acid called sulfanilamide fragment is included in the composition of vitamin Bc instead of natural aminobenzoic acid, which leads to the appearance of pseudofolic acid (Figure 4, XI). This disabled vitamin cannot become a full-fledged metabolite for the biosynthesis of nucleic acids and proteins of the microorganism, which is the basis of the specific bacteriostatic and bactericidal action of sulfanilamides [54,63,64,65,66].

## 5. Modeling of the Mechanism of *o-*Benzoic Sulfimide Antibacterial Action

The theoretical analysis suggests that the antibacterial effect of saccharin is directly related to the isothiazolidine heterocyclic ring that has the ability for biogenic transformation into an activated form in pro- and eukaryotic cells [67,68,69]. This modification may be due to the chemical and/or enzymatic lability of substituted thiazolidine rings.

**Figure 5:**
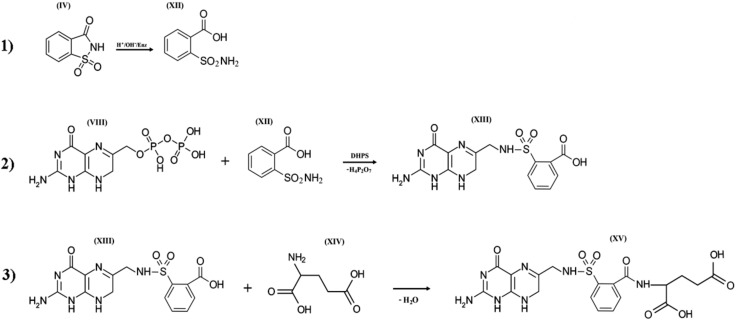
Three hypothetical stages of pseudofolic acid formation based on activated saccharin (IV). Stage (**1**), formation of *o-*sulphamoylbenzoic acid (XII); stage (**2**), interaction of 2-amino-4-hydroxy-6-pyrophosphoryl-methylpteridine (VIII) and (XII) with the formation of an intermediate (XIII); stage (**3**), interaction of (XIII) and L-glutamic acid (XIV) with the formation of a homologue of pseudofolic acid (XV).

When saccharin enters bacterial cells or the human body, it is exposed to extreme pH values [70,71,72] and the influence of cytochrome P-450 enzymes. These enzymes catalyze the oxidation of compounds with aliphatic or aromatic rings, such as the isothiazolidine ring of *o-*benzoic sulfimide (IV), which undergo decyclization to form a compound called *o-*sulphamoylbenzoic acid (XII), which is a structural analog of the antibacterial compounds of the sulfonamides class. In other words, saccharin has antibacterial activity only if it is transformed by the enzyme systems of micro- and/or macroorganisms. *o*-sulphamoylbenzoic acid can be absorbed by both symbiotic and pathogenic microflora. It starts to compete with native *p*-aminobenzoic acid in the bacterial cell and is mistakenly included by the bacterial enzyme complexes in the composition of pseudofolic acid (Figure 5, XV), which cannot provide normal biosynthesis of nucleic acids and many bacterial proteins. This process of pseudofolic acid synthesis passes through the interaction of the phosphated form of 2-amino-4-hydroxy-6-pyrophosphoryl-methylpteridine and *o*-sulphamoylbenzoic acid (Figure 5, stage 2) with the formation of an intermediate, and then one or more l-glutamic acid molecules are attached to it at the final stage (Figure 5, stage 3).

## 6. The Problem of Bacterial Resistance to Sulfonamides

Sometimes parallels between the systems of bacterial resistance to antibiotics and sulfonamides are drawn. In our opinion, this is not entirely correct. For example, the most odious enzymes involved in the utilization of beta-lactam antibiotics are plasmid-encoded β-lactamases.

Current phylogenetic analyses have estimated the age of plasmid-encoded β-lactamases appearing millions of years ago [73,74,75].

A metagenomic DNA study from 30,000-year-old permafrost sediments east of Dawson City, Yukon (Canada) deduced amino acid sequences with up to 84% identities to the modern β-lactamases [75,76]. If we consider the system of resistance to sulfonamides, then only a few studies have shown that some monooxygenases of Microbacterium sp. CJ77 cleavage the sulfonamides [77]. The main bacterial resistance to sulfonamides predominantly occurs because of mutations in *folP* gene encoding dihydropteroate synthase (DHPS) involved in nucleotide biosynthesis or through acquisition of alternative DHPS genes (sul1, sul2, sul3, and sul4), the products of which may have low affinity to sulfonamides [77].

The *folP* gene is present in the genomes of both bacteria and archaea [78], which indicates that both this gene and the mechanism of folate biosynthesis itself probably originated in the ancestral forms of all prokaryotes, i.e., at least 3 billion years ago.

It is obvious that the DHPS is a representative of a much older, vital, and stable prokaryotic folate biosynthetic pathway, in contrast to the β-lactamases. In particular, for this reason, the folate pathway continues to be a promising target for the development of new drugs for antimicrobial therapy. However, there are a number of side effects that require a more balanced approach in the development of new sulfonamide drugs.

These side effects include a non-specific effect on the normal microflora of the gastrointestinal tract and off-target effects of sulfonamide antibiotics on a eukaryotic enzyme, sepiapterin reductase, causing alterations in neurotransmitter synthesis [79].

## 7. Conclusions and Perspectives

*o*-Benzoic sulfimide or saccharin has been known for over a century as a chemical compound and has been used in the food industry as a sugar substitute for more than half a century. Saccharin has passed the test of time, during which it became clear that concerns about its carcinogenicity and teratogenicity are exaggerated, as it is approved of by the Joint FAO/WHO Expert Committee on Food Additives (JECFA) and the European Commission Scientific Committee on Food (CS/ADD/EDUL/148-FINAL February 1997) and is permitted for use in more than 90 countries.

Evidence indicates that the spectrum of properties of this substance is wide and includes antibacterial activity. Until recently, the mechanism of the antibacterial action of saccharin was not characterized, so this article attempted to systematize the available information on this topic and suggest a possible mechanism of the effect of *o*-benzoic sulfimide on the vital systems of bacterial cells.

There is a definite similarity between the mechanisms of the antibacterial action of saccharin and sulfa drugs, and the therapeutic target in both cases should be folic acid of the microbial cell. However, saccharin can only act as a prodrug. Undergoing biogenic transformation, it is able to transform into *o*-sulphamoylbenzoic acid, which is a structural and, apparently, functional analog of several groups of remedies called sulphonamides.

The growing emergence and spread of pathogens resistant to antimicrobial drugs, especially to their natural variants, are a growing concern. This reflects the relevance of the search for new antibacterial therapeutics with rare functional groups or with a non-natural core structure, since the resistance of microorganisms to these develops much more slowly [80,81,82,83]. New data on the antimicrobial action of *o*-benzoic sulfimide indicates the prospects of this compound as a basis for creating a whole group of new antibacterial isothiazolidine derivatives for the pharmaceutical, food, and agricultural industries.
